# PReS-FINAL-1004: Can the cd4/cd8β ratio be used as a predictive biomarker in extended-to-be oligoarticular JIA?

**DOI:** 10.1186/1546-0096-11-S2-P2

**Published:** 2013-12-05

**Authors:** S de Leeuw, B Thirugnanabalan, H Varsani, F Warden, N Makengo, J Lewis, A Pesenacker, L Wedderburn

**Affiliations:** 1 Rheumatology department, UCL Institute of Child Health, London, UK; 2 Life Sciences, HAN University of Applied Sciences, Nijmegen, The Netherlands; 3 immunobiology department, UCL Institute of Child Health, London, UK

## Introduction

Predicting disease course in Juvenile Idiopathic Arthritis (JIA) is difficult. During the first 6 months of oligoarticular JIA (O-JIA) disease presents with 4 or fewer affected joints, however after 6 or more months severity might increase with more than 4 joints involved (extended O-JIA), or persist with 4 or fewer joints affected (persistent O-JIA). However patients do not show any clinical differences at onset making a decision on treatment strategy difficult before extension occurs. It has previously been shown that the CD4/CD8 T cell ratio in extended-to-be patients (samples before extension has occurred) is lower compared to those who persist (Hunter et al 2010), and thus might be a useful biomarker in predicting the course of disease. However measuring this cell ratio currently requires specialized expertise. To translate the measurement of the CD4/8 synovial T cell ratio as a biomarker a method is needed, which could be used in a wide range of hospital facilities, hence a real-time quantitative PCR (qPCR) method was tested.

## Objectives

The aim of this study was to measure CD4 and CD8 mRNA in whole synovial fluid collected at therapeutic joint injection and to compare it to the gold standard of flow cytometry using mononuclear cells.

## Methods

A total of 40 healthy adult blood and 20 patient synovial fluid (SF) samples were included in this study. cDNA was generated from total RNA extracted from Tempus vacutainers using whole blood or SF. CD4, CD8b and GAPDH transcripts were measured by qPCR. Simultaneously peripheral blood mononuclear cells (PBMC) and SFMC were isolated using density gradient centrifugation and stained with CD3, CD4, CD8β and CD8α for flow cytometry.

## Results

Validating previous results, the CD4/CD8 T-cell ratio measured by flow cytometry was significantly lower in SFMC than in healthy adult PBMC. Measurement of CD4/8 ratio by qPCR and flow cytometry showed some correlation in healthy adult PBMC. However CD4/CD8 ratios by qPCR were higher in SF samples compared to healthy control PBMC. This was driven by increased CD4 measurements by qPCR in SF samples.

## Conclusion

Taken together these data show a trend to a correlation of qPCR and flow cytometry methods in healthy adult control samples. The increased level of CD4 transcript in SF measured by qPCR might well be due to the abundance of neutrophils and monocytes, which are/may be discarded during preparation of SFMC for flow cytometry. Whether qPCR measurement of CD4/CD8 ratio can be used as a predictive biomarker for severity of disease course in O-JIA remains to be seen, and will be assessed once clinical follow-up data is available.

## Disclosure of interest

None declared.

